# 5-Hydroxymethylfurfural (HMF) levels in honey and other food products: effects on bees and human health

**DOI:** 10.1186/s13065-018-0408-3

**Published:** 2018-04-04

**Authors:** Ummay Mahfuza Shapla, Md. Solayman, Nadia Alam, Md. Ibrahim Khalil, Siew Hua Gan

**Affiliations:** 1 Laboratory of Preventive and Integrative Bio-medicine, Department of Biochemistry and Molecular Biology, Jahangirnagar University, Savar, Dhaka, 1342 Bangladesh; 2 Department of Biochemistry, Primeasia University, Banani, 1213 Bangladesh; 3 School of Medical Sciences, Universiti Sains Malaysia, 16150 Kubang Kerian, Kelantan Malaysia; 4 School of Pharmacy, Monash University Malaysia, Jalan Lagoon Selatan, 47500 Bandar Sunway, Selangor Malaysia

**Keywords:** 5-Hydroxymethylfurfural, Mutagen, Carcinogen, Antioxidant, Anti-allergen

## Abstract

An organic compound known as 5-hydroxymethylfurfural (HMF) is formed from reducing sugars in honey and various processed foods in acidic environments when they are heated through the Maillard reaction. In addition to processing, storage conditions affect the formation HMF, and HMF has become a suitable indicator of honey quality. HMF is easily absorbed from food through the gastrointestinal tract and, upon being metabolized into different derivatives, is excreted via urine. In addition to exerting detrimental effects (mutagenic, genotoxic, organotoxic and enzyme inhibitory), HMF, which is converted to a non-excretable, genotoxic compound called 5-sulfoxymethylfurfural, is beneficial to human health by providing antioxidative, anti-allergic, anti-inflammatory, anti-hypoxic, anti-sickling, and anti-hyperuricemic effects. Therefore, HMF is a neo-forming contaminant that draws great attention from scientists. This review compiles updated information regarding HMF formation, detection procedures, mitigation strategies and effects of HMF on honey bees and human health.

## Introduction

Honey is a sweet natural product produced by honeybees (*Apis mellifera*), which gather nectar from flowers before converting it to nutritious food. Honey is mainly composed of water (15–20%) and two sugars (dextrose and levulose), with the presence of small amounts of at least 22 other more complex sugars (80–85%, w/w) [41, 179]. Honey has also been reported to contain an intricate mixture of nitrogenous compounds, lactone, proteins, antibiotic-rich inhibine, enzymes, phenol antioxidants, aroma compounds, amino and organic acids, gluconic acid, phenolic acids, flavonoids, minerals, vitamins, 5-hydroxymethylfurfural (HMF) and other phytochemicals [20, 56, 181, 183]. Honey composition varies depending on its floral, geographical and entomological sources [13, 56]. In addition, external features such as seasonal and environmental factors, honey processing, and storage time and conditions have crucial effects on honey’s composition [58, 76, 104].

Honey is considered both nutritional and medicinal, although the presence of certain constituents, for example, heavy metals (even in trace amounts), some alkaloids, and HMF and its derivatives may contribute to honey’s toxicity [77, 152]. HMF is a cyclic aldehyde produced by sugar degradation through the Maillard reaction (a non-enzymatic browning reaction) during food processing or long storage of honey [102]. The presence of simple sugars (glucose and fructose) and many acids, as well as minerals, in honey can further enhance the production of this substance [92]. HMF concentration is widely recognized as a parameter affecting honey freshness because it is typically absent (or is present in only very small amounts in fresh honeys), while its concentration tends to rise during processing and/or because of aging. Previous studies have reported that honey stored at low temperatures and/or under fresh conditions has low or minimal HMF concentrations, while aged and/or honey stored at comparatively higher or medium temperature has high HMF concentrations. In addition to storage conditions, the use of metallic containers and honey floral sources are critical factors affecting HMF levels (Table 1). Hence, higher HMF concentration is indicative of poor storage conditions and/or excess heating of honey [48, 81]. Therefore, the Codex Alimentarius Standard commission has set the maximum limit for HMF in honey at 40 mg/kg (with a higher limit of 80 mg/kg for honeys originating from tropical regions) to ensure that the product has not undergone extensive heating during processing and is safe for consumption [6].

**Table 1:** Variation in HMF concentration in honey samples based on their storage time and geographical sources

Country	Storage time	Storage temperature (°C)	HMF concentration (mg/kg)	References
Asia				
Bangladesh	> 1.5 years	20–25	3.18–703.10	[76]
India	Fresh	–	0.15–1.70	[160]
Malaysia (year)	< 1	4–5	0.26–68.99	[108]
	> 1	25–30	206.06–383.39	[81]
	> 2	25–30	986.57–1131.76	
Nepal	–	25–29	30.36–56.10	[131]
Pakistan	–	4–5	ND–6.00	[3]
	–	–	23.18–27.37	[74]
Iran	–	–	0.04–17.20	[104]
Turkey	Fresh	–	0.00–11.50	[188]
	< 6 months	–	19.20–28.6	[91]
	1 year	20 ± 5	8.60–39.00	[188]
Europe				
Italy	Fresh	–	1.23–5.95	[47]
Croatia	–	–	0.00–23.69	[173]
Czech Republic	4 years	20 ± 2	10.30–44.20	[80]
Poland	–	–	0.70–3.50	[89]
Portugal	–	Room temperature	18.00–94.00	[66]
Spain	< 3 years	–	0.00–21.39	[116]
Northwest Spain	< 2 years	− 30	0.00–1.60	[120]
Switzerland	< 7 years	4	0.00–112.00	[148]
Africa				
Algeria	< 1 year	4–6	1.73–480.00	[36, 82]
Burkina Faso	< 1 year	0–4	3.00–27.50	[103]
Ethiopia	< 6 months	–	0.68–6.56	[25, 85]
Kenya	< 1 year	25 ± 2	3.70–389.36	[112]
Morocco	–	4	3.20–52.60	[169]
Nigeria	< 6 months	–	0.66–1.43	[16]
Tanzania	–	–	5.00–26.40	[115]
South America				
Argentina	–	23 ± 2	0.05–2.94	[21]
	Within 2 months	–	1.48–34.08	[34]
Argentinean Patagonia	> 3 years	–	0.00–14.70	[8]
Brazil	–	–	1.50–115.20	[5, 162]
North America				
Cuba	< 1 year	4	3.30–15.90	[9]
Mexico	–	Room temperature	9.01–21.96	[106]
Australia				
Australia	2 years	− 18	1.30–12.40	[71]

*ND* not detected, *HMF* 5-hydroxymethylfurfural

HMF is not only present in honey; it is nearly ubiquitous in our daily heat-processed, sugar-containing foodstuffs, from our breakfast cereals, breads, dairy products, and fruit juices to liquors at different concentrations [17, 37, 114, 137, 155, 170]. Therefore, HMF is considered one of the main quality indexes of different commercial whey proteins, molasses and many other products [40]. In most previous studies, HMF has been reported to have negative effects on human health, such as cytotoxicity toward mucous membranes, the skin and the upper respiratory tract; mutagenicity; chromosomal aberrations; and carcinogenicity toward humans and animals [59, 94, 107]. However, in more recent extensive studies, HMF has been proved to have a wide range of positive effects, such as antioxidative [193], anti-allergic [187], anti-inflammatory [86], anti-hypoxic [96], anti-sickling [1], anti-hyperuricemic effects [97].

It has been reported that humans may ingest between 30 and 150 mg HMF daily via various food products; however, safe levels of HMF consumption are not well clarified. The reason is that HMF’s metabolism, biotransformation and excretion and thus clearance rate from the body depend on the organ function of an individual [176], which have not been considered. The aim of this review is to describe the effects of HMF present in honey, which, if broadly analyzed, can be used to promote more widespread application of honey with special medicinal implications. Upon highlighting the HMF content in honey, a general discussion of the HMF content in other foods and HMF’s detection, optimized formation, mitigation and eradication from food is provided. For this purpose, all related articles relevant to the topic of “honey and other foods, HMF: its toxicity and therapeutic effects” were included, regardless of the time of publication. To our knowledge, this is the first study to extensively report on HMF in honey from all over the world and to provide a general overview to explore its effects on both bee and human health.

## Formation of HMF in honey

**Fig. 1:**
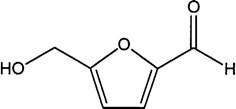
Chemical structure of HMF

HMF is a six-carbon heterocyclic organic compound containing both aldehyde and alcohol (hydroxymethyl) functional groups. The ring of the structure is centered on furan moieties, whereas the two functional groups, i.e., formyl and hydroxy-methyl groups, are linked at the second and fifth positions, respectively (Fig. 1). HMF is a solid, yellow substance that has a low melting point but is highly soluble in water [142].

**Fig. 2:**
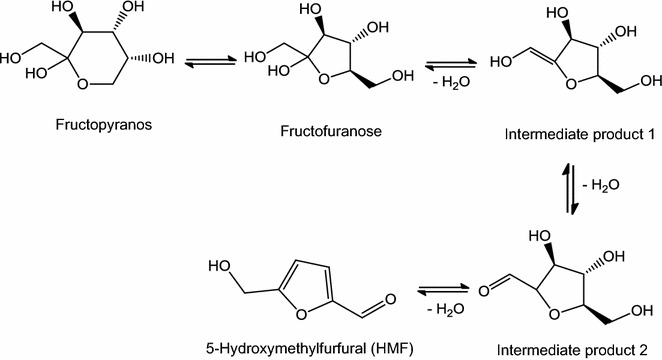
Formation of HMF in honey

HMF is considered the most important intermediate product formed during two reactions (i) acid-catalyzed degradation of hexose and (ii) decomposition of 3-deoxyosone in the Maillard reaction (Fig. 2) [46]. HMF formation is correlated with chemical characteristics such as pH, free acid content, total acidity, lactone content and mineral content, which in turn are related to the floral source of collected honey samples. The presence of simple sugars such as glucose and fructose and of many acids has been reported to be favorable for honey production [47].

Similarly to honey, which is rich in glucose and fructose, most sugar-containing foods also contain HMF [71]. Nevertheless, although HMF occurs at very low concentrations and can even be absent in both fresh honey and food products, heat treatment and/or prolonged storage conditions can enhance further HMF production. Moniruzzaman et al. [108] reported the mean HMF concentration in Malaysian honeys stored for 2 months at 4–5 °C to be 35.98 mg/kg. In contrast, Khalil et al. [81] found that HMF concentrations in Malaysian honey stored at 25–30 °C for more than a year could reach very high levels (118.47–1139.95 mg/kg). [76] also observed high HMF levels (3.18–703.10 mg/kg) in honey samples from Bangladesh stored for more than a year at room temperature (20–25 °C). Therefore, HMF level is not only indicative of honey freshness but also of storage duration and conditions (Table 1).

## Factors affecting HMF formation in honey

In addition to being directly produced when heating sugar from the degradation of hexoses under acidic conditions at high temperatures and/or during the Maillard reaction [15], HMF is produced from the oligo- and polysaccharides that can yield hexoses upon hydrolysis. However, HMF appears to be more selectively produced from keto-hexoses such as fructose [141]. Interestingly, there are two reasons why higher yields of HMF are obtained from fructose (ketose) than from glucose (aldose). First, the reactivity of glucose is lower than that of fructose, with a lower enolization rate [92]. Enolization is believed to be the rate-determining step of HMF production. Second, fructose forms an equilibrium mixture of difructoses and dianhydrides and thus internally blocks most reactive groups, leading to the formation of certain by-products. In contrast, glucose forms true oligosaccharides that still contain reactive reducing groups, posing a higher risk for cross-polymerization with the reactive intermediates, including HMF [92].

Honey has been reported to contain many different types of sugars. In fact, a recent review by Solayman et al. [163] indicated that honey contains approximately 39.44% fructose, 28.15% glucose and 3.19% sucrose. The cyclic aldehyde HMF is also produced in honey by the degradation of related sugars [81]. Heating of honey during its processing reduces its viscosity, which can prevent crystallization or fermentation [161]. In addition to heating, several other factors influence the formation of HMF in honey, such as honey’s physicochemical properties (pH, free acid content, total acidity, lactone content and mineral content), water activity (a_w_), the use of metallic containers [182], and thermal and photochemical stress [164].

HMF is easily formed at low temperatures in the presence of low-pH or acidic conditions [93], while high temperature and long storage duration increase its concentration to a large extent. Nevertheless, a different pathway is proposed in dry and pyrolytic conditions under which HMF is formed from fructose and sucrose. In addition to temperature and pH, the rate of HMF formation in honey is also dependent on honey’s moisture content [62, 63]. Therefore, many steps are taken to maintain low moisture content in honey samples, including gamma irradiation and heat treatment to inhibit HMF formation.

The rate of HMF formation is also dependent on the fructose:glucose ratio and the type of sugars formed because it has been reported that at pH 4.6, fructose has five times more reactivity than glucose, and a high fructose:glucose ratio will accelerate the reaction [93]. Turhan [175] showed that temperature and duration of heat treatment may both affect HMF formation in honey samples. Moreover, it was shown that heating honey samples collected from Anatolia in Turkey at 135 °C for 100 s produced similar amounts of HMF as that yielded by heating samples to 150 °C for 40 s. According to [150], there is a logarithmic relationship between the storage time and HMF levels in honey. In their study on Malaysian honey samples [81], showed that honey samples stored for 3–6 months had HMF values below the International Honey Commission (IHC) limit for tropical honey (< 80 g/kg); however, samples stored for 12–24 months had HMF concentrations above the recommended level.

Khalil et al. [81] investigated the correlation between the physicochemical properties of honey and HMF formation and found that there was a strong correlation between free acids and total acidity, while there was only a moderate correlation between pH and lactones. In another study conducted on four honey samples (eucalyptus, sulla orange and chestnut), Fellico et al. [47] reported a relationship between HMF concentration and heating time, acidity and pH. A similar relationship was indicated in another study [30], which reported a proportional increase in HMF formation with an increase in temperature by one degree, moisture content and water activity. In addition, the concentrations of metallic ions such as manganese, zinc, magnesium, and iron(II) present in honey have also been reported to have a positive effect on HMF formation [122].

## HMF in foods correlates with urine metabolites

Following oral or intravenous administration, HMF is mainly metabolized into three major metabolites: 5-hydroxymethylfuroic acid (HMFA), 2,5-furandicarboxylic acid (FDCA) and 5-(hydroxymethyl)-2-furoyl glycine (HMFG), with the possible formation of a fourth metabolic product termed 5-sulfoxymethylfurfural (SMF) [67]. However, studies designed to investigate the distribution of radio-labeled [U-^14^C]-HMF and its metabolites by whole-body autoradiography have revealed that neither HMF nor its metabolites are accumulated. It is estimated that 80–100% of the total amount of radioactivity is released within the first 24 h following its administration [55, 61]. HMF is first oxidized to carboxylic acid and is conjugated with glycine, leading to the formation of HMFG. The concentration of free glycine is the rate-limiting step in this pathway. Subsequently, HMF undergoes further oxidation to yield FDCA.

In an in vivo study, [61] reported the presence of three metabolites in urine following oral administration of labeled HMF (10–500 mg/kg), where the relative amounts of FDCA, HMFA, and HMFG excreted were 2–6, 78–85 and 5–8%, respectively (depending on the species and the doses). In humans, HMF is completely cleared following its oral administration (using the juice of dried plum) [130]. Similarly, in another clinical study by Hardt-Stremayr et al. [67], HMF was shown to be completely excreted through urine within 48 h following oral administration at 240 mg/day, while SMF was not detected in urine, as also reported in other studies.

## HMF in various food products

In addition to being found in honey, HMF is also present in dried fruits (> 1 g/kg), products containing caramel, instant coffee (up to 6.2 g/kg), apple juice, citrus juices, beer, brandy, milk, breakfast cereal, baked foods, and tomato products, and HMF is released from sugar and carbohydrates after home cooking, indicating that HMF is ubiquitous in the diet.

We consume several different food products, including bakery goods, milk, fruit juice, cereals, coffee, chocolate, soft drinks, vinegar, wine, nuts and grilled meat in our daily lives. The majority of these produces undergo thermal treatments prior to consumption, such as boiling, baking, extrusion cooking, roasting, pasteurization and other processing. These processes are performed not only to make the products more edible but also 1) for preservation (by means of reducing microbial load and/or eliminating enzymatic activities) and 2) to generate more desirable sensory (color, aroma and taste) and texture properties. Nevertheless, extensive processing produces adverse effects by introducing undesirable, harmful and non-nutritive compounds or by reducing nutritive value, fresh appearance and taste. During thermal processing and preservation, the Maillard reaction or non-enzymatic browning may also occur, where HMF is a common product whose extent of formation depends on the processing and preservation conditions [87].

### Sugar

Sugar is a disaccharide composed of fructose and glucose. It is produced exclusively from sugar beet and sugar cane. Although the extraction and purification processes are very simple, HMF is formed as a result of heat. Polovková and Šimko [128] analyzed brown (n = 25) and white sugars (n = 13) from local markets in the Bratislava territory, the capital city of the Slovak Republic. Upon preparing the sugar samples, HMF levels were determined by high-performance liquid chromatography coupled with a diode array detector (HPLC-DAD) at 284 nm. Surprisingly, white sugar was free of HMF, but brown sugar was found to contain HMF (0.17–6.45 mg/kg). The presence of HMF in brown sugar may be due to the addition of treacle to the preparation preserved at 50 °C to maintain its liquidity. Similarly, using HPLC, Risner et al. [139] found similar ranges of HMF concentration (11.9–16.4 and 12.3–23.3 mg/kg) for light and dark-brown sugars, respectively.

### Cereals

According to Norwegian and German researchers, cereals and cereal products, including bread, are some of the most prominent sources of human exposure to HMF [2, 73]. The extent of HMF formation in cereal products is heavily dependent on many factors, including temperature, dough fermentation process, water activity and the presence of fruits, grains and other flavorings or additives (such as cocoa, malt, sucrose, glucose, salt, and honey).

Mańkowska et al. [101] investigated the HMF concentrations in 41 food products. Wheat bread with cranberries was reported to contain the highest amount of HMF (210 mg/kg), followed by breakfast cereals, i.e., honey wheat loops (85.09 mg/kg). The lowest amount of HMF was reported to occur in gluten-free sponge cakes and whole-grain oatmeal. Sweetened breakfast cereals contained HMF at 25.55 mg/kg, which was higher than the mean HMF concentration (18.40 mg/kg) in bakery products. In another study, the mean HMF concentrations in cereals, i.e., mixed grains (240 mg/kg), cornflakes (7–114 mg/kg) and wheat-based cereals (6–132 mg/kg), were also determined to be high [144, 145]. The products for which the dough fermentation process and the types of flour were specifically affected were again reported to be bread and other bakery products, including cookies. Additionally, HMF concentrations in different types of flours, including wheat, whole wheat, bread flour and rye flour, were investigated. The HMF concentration in bread made with rye flour was determined to be the highest (mean 26.88 mg/kg), perhaps due to the high amino acid content.

Different types of dried fruits (raisins, cranberries, palms, strawberries, red currants and apple) are added to cereal products, which normally contribute to high HMF concentrations. In fitness and rice-wheat flakes with raisins added, the HMF concentrations were reported to be 6.78 and 11.70 mg/kg, respectively. The lowest (6.06 mg/kg) HMF concentration was reported to occur in crunchy products containing raisins and plums. On the other hand, red fruits such as apples, strawberries and red currants confer some of the highest concentrations of HMF to food products, including oatmeal (47.62 mg/kg) [101]. Similarly, bread containing dried fruits tend to have higher HMF concentrations than does white bread [137, 156], indicating that the main contributor of HMF is dried fruits.

### Coffee

Coffee is one of the most common drinks reported to contain HMF. The HMF concentration in coffee depends on the brewing processes or types of coffee (mocha, espresso, filtered coffee or plunger-brewed coffees) used, as well as the amount of sugar added to it. Mortas et al. [111] investigated HMF concentrations in Turkish coffees (either prepared traditionally or of the instant variety) using HPLC coupled with a diode array detector. The authors reported that before brewing, instant and traditional Turkish coffee samples contained HMF over ranges of 336.03–362.05 and 213.02–238.99 mg/kg, respectively. However, following brewing, the HMF concentration increased by 32.29–55.83% (in instant coffee) and 74.12–224.75% (traditional coffee), respectively.

Arribas-Lorenzo and Morales [15] used reversed-phase HPLC coupled with UV detection to detect HMF concentrations in three types of ground coffee consumed by the Spanish and found significant differences in the concentrations observed. HMF levels were 110 mg/kg (natural coffee: produced by traditional roasting of coffee beans), 625 mg/kg (torrefecto coffee: obtained by adding sucrose prior to the roasting process) and 1734 mg/kg (blended coffee: a combination of natural and torrefacto ground coffee in various proportions). However, soluble coffee contained the highest level (2480 mg/kg). The authors concluded that the daily HMF intake of a high coffee consumer in Spain is approximately 122.42 μg/kg, indicating that heat contributes to HMF formation.

### Dairy products

HMF is formed via side reactions during heat sterilization and browning processes. Albalá-Hurtado et al. [4] investigated the formation of HMF as a result of exposure to different storage temperatures (20, 30, 37 °C) and storage duration (up to 9 months) in liquid as well as in powdered infant milk. They found that HMF formation follows a zero-order kinetics profile independent of storage temperature and milk type. A powdered infant milk sample stored at 37 °C for 12 months contained higher amounts of HMF and furfural compounds (31.5 µmol/L) than those present in liquid milk (2.5 µmol/L).

In another study on ultra-high temperature processed (UHT) milk, no significant variation in HMF levels was observed for samples stored at 4 and 8 °C. However, storage at room temperature led to a two-fold increased formation of HMF [33]. In the case of traditional Indian dairy products, there was a strong positive correlation between HMF concentration and the products’ their flavors, colors and textures [70]. Morales and Jiménez-Pérez [110] used micellar electrokinetic capillary chromatography and reported a mean HMF concentration of 29.5 µg/kg for many infant milk-based formulas.

### Fruits and vegetables

Due to their rich content of sugars and amino acids, fruits and vegetables contain high HMF levels. In a study involving jam products (prepared commercially and under laboratory conditions) stored at 20 and 35 °C for 12 months, a temperature-dependent relationship was established between HMF formation and storage duration [133]. Additionally, a positive correlation between storage time and temperature with HMF formation has been observed for two varieties of apple juice (Golden amasya and delicious) [32]. Ordóñez-Santos et al. [121], who investigated changes in HMF levels in bottled tomato puree stored at 20 °C for 180 days, reported a negative correlation between HMF formation and the content of organic acids such as ascorbic, citric and mallic acids.

Murkovic and Pichler [114] analyzed HMF concentrations in dried apricot, peach, pear, fig, date, apple and pineapple products. HMF concentrations were highest in dates (1000 mg/kg) and plums (1100–2200 mg/kg). The mean range of HMF concentrations in other dried fruits was 1–780 mg/kg [114]. On the other hand, in a study by Rufían-Henares et al. [146] on commercially dehydrated vegetables, HMF was not detected, except in cabbage, tomato and artichoke (58.60, 18.20 and 6.97 mg/kg respectively).

Oil concentration in products may also affect HMF formation. To investigate this hypothesis, Fallico et al. [45] roasted defatted and ground hazelnuts containing different amounts of hazelnut oils or oil containing saccharose and/or hexanol. The defatted hazelnuts roasted in high oil concentrations exhibited increased HMF formation. Non-defatted hazelnuts with saccharose contained the highest HMF concentration (372 mg/kg), while defatted hazelnuts with saccharose contained the lowest (33.5 mg/kg). Because the HMF concentration increased from 66.5 to 144.0 mg/kg in non-defatted samples upon prolonged roasting (from 30 to 60 min respectively), the authors also concluded that HMF levels increased with the duration of the heat treatment applied to the products.

### Other miscellaneous food products

It is concluded that there are no heat-processed food products that are free of HMF. The HMF concentrations in several food products are listed in Table 2.

**Table 2:** HMF concentrations in different food products

Food type	HMF concentrations (mg/kg)	Detection method	Country	References
Must syrup	3500–11,000	GC–MS	Spain	[147]
Molasses	100			
Sugarcane syrup	100–300			
Palm syrup	< 3			
Prunes	237	HPLC	Norway	[73]
Dark bear	13.3			
Canned peaches	5.8			
Raisins	5.0			
Balsamic vinegar	316–3250	HPLC	Italy	[171]

*GC–MS* gas chromatography–mass spectrometry, *HPLC* high performance liquid chromatography

### Comparison of HMF levels in honey and different food products

HMF is not present naturally in food products. The compound is formed upon thermal treatment and in combination with other factors. As it is a product of the non-enzymatic Maillard reaction, there is no fixed concentration of HMF in different food items. The baking temperature, rate of saccharose degradation, concentration of reducing sugars, type of sugar (glucose, fructose or others), water activity, the addition of other food additives such as HMF-containing sweeteners, coloring agents, caramelization, storage time and temperature, type of metallic storage and processing container vary widely among different food items. Therefore, HMF content varies among food items, even among those of the same type. Nevertheless, honey is a safer food item than other processed foodstuffs with respect to its HMF concentration. Turhan et al. [174] showed that the initial heating temperature and time are not directly correlated with HMF concentration in honey. Nectar honey and honeydew honey processed at 95 °C for 90 min and 90 °C for 75 min showed HMF levels lower than 40 mg/kg. Storage temperature and storage duration in particular directly influence the development of HMF in stored honey [44, 81]. Unlike for honey, in the processing of other foodstuffs, comparatively higher temperatures (during baking, roasting), longer duration times, and different additives are required, which profoundly affect the HMF content in the foods. For example, cookies baked at high temperature contain 10–100 times more HMF (167.4–1100.1 mg/kg) than cookies baked at 200 °C (9.9–39.6 mg/kg) [10]. Fresh cookies baked at 300 °C and with saccharose added during processing have been reported to contain as much as 1100 mg/kg HMF. Even ammonium bicarbonate addition may dramatically increase the HMF content (above 3500 mg/kg) of saccharose-containing cookies baked at 220 °C. In investigating 22 coffee samples, Murkovic and Pichler [114] reported HMF concentrations ranging from 300 to 1900 mg/kg. Murkovic and Bornik [113] found that HMF formation was dramatically increased in coffee roasted at 240 °C within the first 3 min, with concentrations reaching up to 900 mg/kg. In the case of bread, HMF values vary widely, from 3.4 to 176.1 mg/kg, depending on the fermentation conditions, the leavening agents added, the thickness of the crust and crumb, and the type of bread (oat bread, white bread or wheat bread) [137]. According to Raimirez-Jimenez et al. [137], the HMF content of biscuits (15.6 mg/kg) is higher than that of confectionaries such as doughnuts and croissants (9.5 mg/kg). Thus, the formation of HMF is inevitable, and comparing or ranking food items with respect to HMF concentration cannot be performed precisely.

## Detection methods of HMF

Accurate quantitative analysis of HMF is of great importance because HMF is a marker of quality deterioration, thermal processing and other adulteration practices. HMF is also important in clinical research and therapeutics. The IHC has recommended three main methods for HMF determination: two spectrophotometric methods by White and Winkler and a reversed-phase high-performance liquid chromatography (HPLC) method [191].

Prior to the availability of the spectrophotometric methods, both optical and chemical methods were used. In his newer spectrophotometric method, White utilized a clarified honey sample containing 0.1% sodium bisulfate as a reference and as honey solution without sodium bisulfate as a sample [180]. On the other hand, the Winkler method utilizes honey solutions with p-toluidine and barbituric acid [184]. Nevertheless, although these methods are fast, they lack good sensitivity and specificity. Moreover, the method described by Winkler requires the use of *p*-toluidine, which is carcinogenic. Although the HPLC method is relatively more expensive, it is advantageous with respect to both labor and time. In addition, the method is deemed as an automated and sensitive method that can exclude many interferences from other related compounds [185]. Nevertheless, although HPLC is a sophisticated technique, the method is still not satisfactory to some who have recommended further development and modification of the method [13].

Reyes-Salas et al. [138] reported an electrochemical approach for HMF detection. In this method, a single and sharp reduction signal was created at − 1100 mV versus argentum or argentum chloride, while borate was used as a supporting electrolyte. Another method is the ion exchange liquid chromatography–photodiode array detection technique described by Yuan and Chen [189], which is consistent with Winkler’s method. Another method involves automated flow injection, as reported by Iglesia et al. [38], which is based on the operating principle of the Winkler method and provides a detection range of 5–40 ppm. Micellar electrokinetic capillary chromatography (MEKC) is another rapid method that uses caffeine as a standard. The technique is suitable for rapid quantification of HMF, particularly in honey samples, without requiring sample pretreatment [140]. A unique and efficient rapid screening technique is direct analysis in real time (DART) coupled with time-of-flight mass spectrometry (TOF-MS), which has been reported to yield a chromatogram with high resolution [134]. The method can quantitatively analyze HMF concentrations in a more precise manner than other methods and requires no (or very little) sample pretreatment.

## Optimization of HMF formation in different foods

HMF is formed unavoidably in most foods containing monosaccharides (such as glucose, galactose, or fructose) during processing as a function of temperature and storage period. The process of HMF formation in any food is multifactorial. Factors affecting the rate of HMF formation include temperature, pH [62], type of sugar [93], concentration of divalent cations [64] and water activity [63, 90] in the medium. Hence, to control and optimize the level of HMF formation is a troublesome task. Therefore, food processors must know the effects of different factors so that necessary precautions can be adopted previously to limit and/or optimize the synthesis of HMF in food. To understand the conditions under which HMF would be formed only at desirable levels, a more fundamental and systematic approach must be adopted. The possible effects of these factors must be studied simultaneously and in combination. Toker et al. [172] recommended central composite face-centered design (CCFD) of response surface methodology (RSM) as a useful experimental technique for this purpose. According to Hunter [72], this versatile and effective systematic tool can be used to determine the optimal levels of the contributing factors for the parameters concerned. The mathematical expression derived by this methodology can be employed to develop predictive models upon setting the levels of the various influencing factors to reach the optimum HMF concentration in food.

## Hazard posed by HMF to honey bees

**Fig. 3:**
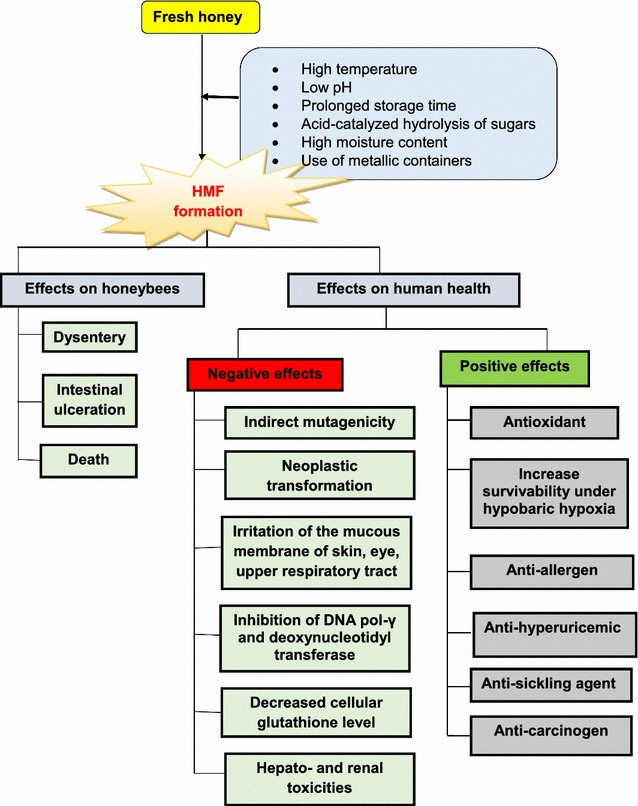
HMF effects on honey bee and human health

The productivity of apiculture is highly dependent on the health, survival and quality of honey bees. Therefore, improving the health quality of bee food is an important priority. Normally, sources of carbohydrates for honey bees are the nectar and honeydew collected from plants, as processed by foragers. However, in the case of commercial honey production, the food and nutrients of cultured bees are depleted following the collection of the autumn yield from hives. Furthermore, honey bees’ natural resources tend to wane during winter. Therefore, bee keepers must use alternative sources to replenish the carbohydrate demand of bees, such as sucrose, high fructose corn syrup (HFCS), various fruit sugars and invert sugars [117]. Among these, however, compared with sucrose, HFCS is considered a dynamic source due to its practical storage, transportation facility and liquidity as opposed to the crystallization method. HFCS is produced commercially from corn starch by using enzymes such as alpha amylase, amylo-glucosidase and invertase [26, 29], whereas sucrose is produced by acid hydrolysis alone [18]. Regardless, these sources are not safe because HMF and its daughter products (formic and levulinic acids) are formed, thus posing a potential threat to honey bees (Fig. 3). Beet sugar, which is also used to feed bees, has been reported to contain up to 475 mg/kg HMF, depending on the processing and storage conditions [177]. In another study, a sugar solution containing 30–150 mg/kg of HMF was used to feed honey bees and was found to cause 15–58.7% of deaths of caged bees within 20 days [78]. Krainer et al. [88] reported that the LD50 values of HMF for bee larvae fed for more than 6 days were 4280 and 2424 mg/kg on days 7 and 22, respectively. Interestingly, they found that 7-day larvae emerging after being fed HMF for 6 days were more sensitive than were adult bees, but the emerging adult bees at day 22 were less sensitive. Moreover, the authors concluded that HMF used as a supplement in food does compromise colony fitness but may not cause great losses of brood. It is hypothesized that HMF causes bees to experience dysentery-like symptoms and ulcers in the gastrointestinal intestinal tract [18], leading to their death.

## Effects of HMF on human health

HMF and other congeners exert both detrimental and positive effects on human health (Fig. 3).

### Adverse effects on human health

HMF and its derivatives have been confirmed to confer genotoxic, mutagenic, carcinogenic, DNA-damaging, organotoxic and enzyme inhibitory effects.

#### HMF as an indirect mutagen

Florin et al. [51] investigated the mutagenic activity of certain compounds, including HMF, towards four mutant strains of *Salmonella typhimurium*. Liver extracts from methylcholanthrene-induced rats were used for metabolic activation of the investigated compounds, and it was confirmed that HMF is not a mutagen. In contrast, Lee et al. [94] demonstrated that HMF is an indirect mutagen because it is converted to an active metabolite, the sulfuric acid ester 5-sulfo-oxymethylfurfural (SMF), with mutagenicity towards *S. typhimurium* TA104. HMF is enzymatically activated to SMF by sulfotransferases (SULT) contained in rat liver extracts enriched with the sulfo group donor 3′-phosphoadenosine-5′-phosphosulfate (PAPS). This finding is further supported by another study in which HMF, 2,5-bishydroxymethylfuran (metabolite of HMF), furfuryl alcohol (FFA) and 5-methyl-FFA showed mutagenicity towards *S. typhimurium* TA100 expressing human SULT1C2 but not towards the parental strains [60].

Another study using FVB/N (FVB) mice expressing hSULT1A1/1A2 revealed that HMF has a genotoxic effect. Mice orally administered single doses of 900 or 1300 mg/kg showed significant DNA damage of their renal cells, as detected by an alkaline single cell gel electrophoresis assay. Another furan derivative, 2,5 dimethylfuran (DMF), also causes DNA damage in the kidney and the colon [69]. In an in vitro erythropoietic micronucleus assay, rat bone marrow cells were exposed to DMF at a concentration of 0.1 mM for 1 h. The study indicated that DMF exhibits genotoxicity toward hematopoietic cells [53].

In another study conducted on five cell lines possessing different levels of SULT1A1 activity (mouse L5178Y, no activity; Chinese hamster: V79-Hp-PST, high activity; V79, negligible activity; human: HEK293, higher activity; and Caco-2, low activity), HMF exerted DNA damage when exposed at a concentration of 100 mM for 3 h [42]. However, it was also determined that HMF poses DNA-damaging effects irrespective of the SULT1A1 activity of the cell lines. In addition, 5-HMF was determined to cause chromosomal aberrations in a Chinese hamster V79-derived cell line constitutively expressing human sulfotransferase SULT1A1 and CYP2E1. In fact, 5-HMF is a potent inducer of sister-chromatid exchange at a concentration of 19.8–3808.0 µM in cells exposed for 32 h [59]. Nishi et al. [118] confirmed that 5-HMF causes chromosomal aberrations of V79 cells at 15.8 mM. In both their pre-clinical and clinical studies, Pastoriza et al. [125] indicated that orally administered HMF is converted to reactive SMF after being absorbed through the gastrointestinal tract. SMF forms a DNA-SMF adduct in mice’s kidney, leukocytes and liver cells, as well as in leukocytes of pre-adolescent populations. In addition, SMF is not properly excreted through urine due to renal reabsorption, thus allowing SMF to accumulate in the plasma, making it available to react with cellular proteins and DNA.

#### HMF as a dual player in carcinogenesis

HMF and its derivative SMF have been confirmed to be potent carcinogens in several studies at the preclinical level. The compounds cause neoplastic transformations in several organs, including the colon and the skin. Colon cancer involves the development of a multistep stage in which micro-adenomas and aberrant crypt foci (ACF) are the morphological markers [14]. HMF acts as an initiator as well as a promoter of ACF [28]. A study was conducted on multiple intestinal neoplasia mice that were subcutaneously administered with a single dose of HMF (500 mg/kg) or SMF (25 mg/kg) within 3–6 days after birth. After 12 weeks, administration of both HMF and SMF increased the number of small intestinal adenomas and flat dysplastic lesions (flat ACF) in the large intestine [168]. The mice had a mutant copy of the tumor suppressor gene adenomatous polyposis coli (*APC*). Mutation in the APC gene leads to the development of adenomas in the small and large intestines, analogously to human familial adenomatous polyposis syndrome (FAP) [127, 129].

In a study by Zhang et al. [192], 45% of F344 female rats administered with HMF at 250 mg/kg twice per day by oral gavaging were found to develop a large intestinal ACF on day 30. HMF increases both the number and size of ACF. In contrast, an extensive study (with two mouse models: wild-type FVB/N and genetically engineered mice having several copies of human SULT1A1 and SULT1A2 at chromosome 9), Florian et al. [50] yielded opposite findings (i.e., neither HMF nor its metabolite SMF induces ACF and colonic tumors). HMF also induces skin papilloma. Upon topical application of sulfoxymethyl and chloromethyl derivatives of HMF, the mice were found to develop papillomas on their skin.

Another derivative of HMF, 5-chloromethyfurfural, has been found to induce hepatocarcinoma in B6C3F1 male rats at a very early age [166]. Schoental et al. [153] showed that rats subcutaneously administered with HMF (200 mg/kg), developed renal lipomatous tumors. In contrast to these studies, a study by Zhao et al. [194] using the A375 cell line indicated that HMF can induce apoptosis and G0/G1 arrest in DNA-damaged cells via the reactive oxygen species (ROS)-mediated signal transduction pathway. Thus, it is concluded that HMF is a potent anti-carcinogen.

#### HMF as an organotoxic agent

HMF has cytotoxic effects at high concentrations, as the compound causes irritation in the mucous membranes, skin, eyes and upper respiratory tract [109]. SMF was revealed to be a strong nephrotoxic agent in an in vivo study in which male FVB/N mice were intraperitoneally administered with SMF (250 mg/kg). After 5–11 days post-treatment, the mice either died or became morbid, possibly due to liver damage or more severe renal damage, particularly at the proximal tubules. A histopathological study revealed extensive proteinaceous casts and necrosis in the affected area [22]. The organic anion transporters type 1 and 2 (OAT1 and OAT2) are highly conserved in different species, including rat and human, and are usually expressed on the cellular basolateral membrane of the proximal tubules. The transporters mediate the uptake of different organic anions, including SMF, from the blood stream as their substrates and are concentrated into tubular cells, which mostly affect the proximal tubules [31]. Another study using human embryonic kidney cells (HEK293) that constitutively express OAT1 and OAT2 transporters supports this mechanism [19]. Glutathione is an important endogenous antioxidant in the body. An ex vivo study involving two mammalian cell lines, V79 and Caco-2, indicated that HMF decreased cellular glutathione levels in a concentration-dependent manner at 50 mM and 120 mM, respectively [79].

#### HMF as an enzyme inhibitor

The human genome encodes 16 DNA-dependent DNA polymerases. Among them, three are involved in nuclear DNA replication, while the remaining are involved in the repair system [24, 52]. Human polymerase γ is a multifunctional enzyme with DNA polymerase, terminal transferase, 5′-deoxyribose phosphate (dRP) lyase and polynucleotide synthetase activities. The primary structure of the polymerase includes a nuclear localization signal, a BRCA1 carboxy terminus domain, a proline-rich region, a pol β-like region and a pol X region [27, 135, 136]. DNA pol γ shares a sequence homology with the terminal deoxynucleotidyl transferase (TdT) that catalyzes the addition of deoxyribonucleotide to the 3’ end of dsDNA or ssDNA in a template-independent manner [124, 143]. HMF competitively inhibits DNA pol γ and TdT with respect to the deoxynucleotide substrate and DNA template primer with 50% minimum inhibitory concentration (IC_50_) values of 26.1 and 5.5 µM, respectively [105].

### Positive effects of HMF on human health

#### HMF as an antioxidant

ROS are produced as toxic by-products of the body’s aerobic metabolism. The species oxidize cellular macromolecules such as proteins, membrane lipids, and DNA and cause cellular damage. The consequences range from stress to metabolic defects, neurodegenerative diseases or even neoplastic transformations [159]. In a study by Zhao et al. [193], HMF showed a dose-dependent (0.8–6.4 mM) free-radical scavenging capacity. HMF also has significant protective effects on erythrocytes against ROS-induced damage. To investigate the protective effect and oxidative stress induced by 2,2′-azobis (2-amidinopropane) dihydrochloride (AAPH), the levels of ROS and malondialdehyde (MDA: indirect determinant of lipid peroxidation) production, the activity of the antioxidant enzymes glutathione peroxidase (GPx), superoxide dismutase (SOD), and catalase (CAT) in erythrocytes pre-treated with HMF were determined. It was revealed that the contents of ROS and MDA were reduced in HMF-treated cells, while the activities of these enzymes were elevated relative to those of negative control cells [193]. The ROS scavenging activity of HMF is imparted by its structure, which features functional reactive groups such as an aldehyde oxygen, double bonds and another oxygen in its furan ring. These features can attract electrons easily and quench ROS [176].

HMF also has a protective effect at the morphological and biochemical levels on hepatocytes damaged by hydrogen peroxide (H_2_O_2_)-induced oxidative stress, under which cells undergo morphological changes such as wrinkling, condensation of chromatin and splitting of nuclei—hallmarks of apoptotic and necrotic cells. However, in a H_2_O_2-_stressed human liver cell line (L02), cells treated with HMF (0.79 µM) could preserve their morphology better than non-treated cells could. HMF also reduces the levels of caspases 3 and 9 (executioner of apoptosis) in these cells [178]. Ding et al. [39] hypothesized the underlying protective biochemical mechanism, which may be due to inhibition of apoptosis by accelerating the transition of cells in the S phase to the G2 or M phase, as well as decreased levels of nitric oxide and caspase 3.

#### HMF against hypoxic injury

Oxygen is required for cell survival. Deficiency of oxygen (hypoxic condition) has numerous detrimental and even life-changing effects on health. Hypoxia may be induced by many factors, including altitude and self-related conditions such as ischemia, atherosclerosis and cancer [126]. Several cellular mechanisms are triggered and can ameliorate hypoxic conditions, among which extracellular signal-regulated kinase(ERK)-mediated transactivation of the transcription factor and hypoxia-inducible factors (HIF) are believed to participate [151]. The mitochondrial membrane potential is also reduced and negatively affects hypoxic cells [75]. In their in vitro study of the cell line ECV304 (human umbilical cord vein endothelial cell), Li et al. [96] showed that cells pre-treated with HMF (200 µg/ml for 1 h) before being exposed to hypoxic conditions (0.3% oxygen for 24 h) exhibited increased mitochondrial membrane potential and decreased phosphorylated ERK levels. The number of apoptotic and necrotic cells also significantly declined. In their further study with a Kunming mice model, the authors showed that pre-exposure to HMF (100 µg/ml, 1 h) significantly attenuates the extent of hypobaric hypoxia-induced permeability of the blood–brain barrier (BBB). Pre-exposure also decreases the extent of neuronal damage in the CA1 region of the hippocampus. Thus, because HMF increases survivability under hypobaric hypoxic conditions, it may be a potent therapeutic agent against acute mountain sickness (AMS), high-altitude cerebral edema (HACE) and high-altitude pulmonary edema (HAPE) [95].

#### HMF as an anti-allergen

Basophils and mast cells participate in the pathogenesis and manifestations of allergic reactions such as asthma, atopic dermatitis and allergic rhinitis. RBL-2H3 cells are mast cells located in the mucosal layer. These cells express immunoglobulin Fc epsilon receptor type I (FcεRI) on their surface. The crosslinking of IgE with specific protein antigens and the binding of crosslinked IgE to FcεRI trigger intracellular signal transduction cascades. These events lead to Ca^2+^ influx and the release of mediators by degranulation, MAPK phosphorylation, upregulation of cytokine gene expression and increased ROS generation [23, 83, 84, 154]. Yamada et al. [187] showed that HMF acts at different stages to inhibit degranulation at doses of 0.01–0.30 µg/ml. In addition, HMF interferes with antigen-antibody crosslinking and antibody-receptor binding. HMF also blocks calcium (Ca^2+^) influx into IgE-sensitized bovine serum albumin-stimulated RBL-2H3 cells. Nicotinamide adenine dinucleotide phosphate (NADPH) oxidase plays a major role in the production of ROS in IgE-mediated RBL-2H3 cells. H_2_O_2_ and NO, two major ROS, are known to regulate degranulation and Ca^2+^ signaling in mast cells [83, 84, 186]. A significant inverse association exists between the release of histamine and Ca^2+^ from intracellular stores and the superoxide anion or DPPH scavenging activities. The anti-allergen effect of HMF on cells is due to the blocking of histamine release and Ca^2+^ signaling through the compound’s free-radical scavenging activity [98, 167].

In another study using ovalbumin (OVA)-immunized BALB/c mice, HMF decreased the levels of total IgE and OVA-specific IgE. The study also showed that HMF-treated immunized mice exhibited lower levels of IFNγ (Interferon gamma) and IL-4 (Interleukin 4) than those of untreated mice. Therefore, HMF may be a potent anti-allergic compound [7].

#### The use of HMF for other pathologic conditions

Uric acid is the end product of purine catabolism. The final two steps of the purine catabolic pathway are catalyzed by a critical enzyme, xanthine oxidase (XO). Uric acid is mainly excreted via urine. High levels of uric acid in the blood lead to the development of hyperuricemia [123], which is the main cause of gout. In addition, many other pathological states are associated with hyperuricemia, including metabolic syndrome, heart failure, pulmonary disorder and type 2 diabetes mellitus [68]. Increased XO activity downregulates the anti-inflammatory transcription factor peroxisome proliferator-activated receptor-γ (PPARγ) and accelerates inflammatory action [57]. XO is also an endogenous producer of superoxide, a potent activator of nuclear factor kappa B (NFκB) [99]. NFκB acts as a transcription factor and upregulates the expression of nitric oxide synthase 2 (NOS2) and interleukin 8 (IL-8). HMF exerts an anti-inflammatory effect by downregulating NFκB [86] and inhibits the activity of XO [97].

#### HMF as an anti-sickling agent

Hemoglobinopathies such as sickle cell disease are life-threatening. The underlying mechanism is polymerization of abnormal hemoglobin (sickle hemoglobin, HbS) under hypoxic conditions. The pathophysiology is manifested by the deformation of red blood cells, loss of resilience and occlusion of tiny blood capillaries, leading to morbidity [65, 100, 165]. Although there are many anti-sickling agents, these agents are not free of side effects. Many agents also exhibit reduced bioavailability in minimal doses and react to non-target proteins. In addition, HMF can act as an effective anti-sickling agent. Abdulmalik et al. [1] showed that HMF orally administered in low concentrations is absorbed into the blood stream from the gastrointestinal tract in transgenic (Tg) sickle mice. Subsequently, HMF can penetrate the erythrocytes and form stable a Schiff-base adduct with the N terminal αV11 nitrogen of HbS in a symmetrical fashion. Following the formation of such an adduct, HMF allosterically shifts the oxygen equilibrium curve to the left and prevents the sickling of erythrocytes. In fact, the authors also reported that Tg sickle mice pre-treated with HMF survived longer under hypoxic conditions than did untreated mice, indicating the potential of HMF as an anti-sickling agent.

## Tolerable daily intake (TDI) of HMF

Many studies have revealed that different cells respond to 5-HM-induced cytotoxicity differently. The susceptibility of cells to HMF depends on the presence and expression levels of receptors [119], metabolism [157], structure [149] and the enzyme activity of HMF [158]. At the preclinical level, no toxic effects have been observed at daily doses ranging from 80 to 100 mg/kg body weight [2]. Zaitzev et al. [190] established the TDI for HMF as 132 mg/day using a 40-fold safety margin. The European Food Safety Authority (EFSA) [43] established a threshold of concern of 0.54 mg/day for the intake of furan derivatives used as flavoring agents in Europe. However, in their study involving 268 Spanish school children, Pastoriza et al. [125] found that although the students’ daily HMF intake, well-distributed throughout the day, was 10–70 mg, a statistically significant level of SMF, a potent toxic metabolite of HMF, was found in their plasma. In addition, most experiments concerning the health effects of HMF have been carried out in vitro, ex vivo and at the experimental animal level. Thus, based on the data available to date, it is not possible to ascertain a TDI. In addition, further research, particularly at the clinical level, paving the way for recommending a TDI for HMF is worth considering and appreciating.

## Mitigation strategies

There is no particular strategy for mitigating the formation of HMF in honey due to the numerous precursors of HMF and the types of reaction orders involved [35]. HMF is formed following a zero-order kinetic process in an exponential manner [54]. Various mechanistic strategies may be adapted to mitigate the formation of HMF. There is a positive relation between a high temperature-time profile and acidic pH for the formation of HMF, which can be modified [62]. In fact, the reduction of thermal input can significantly decrease the formation of HMF, which can be achieved by various means, for example, by adjusting the temperature of the processing oven when the moisture content is high or, conversely, by decreasing the temperature when the moisture content is low. Reduced thermal input can also be achieved by heating food to a low temperature for a prolonged period by vacuum frying or by dielectric heating (microwave and radiofrequency). In the case of dielectric heating, an external alternating current is applied, causing the dielectric molecules in food products to undergo rotation and friction, thus ensuring the rapid generation of uniform heat. In addition, because water molecules are the main target of this mechanism, heat is generated in the portion of food containing water [195]. Because long storage periods lead to high HMF formation in honey [81], shortening the storage period can reduce the formation of HMF.

## Strategy for removing HMF and other furans

To remove already formed HMF, a post-process vacuum technology can be exploited. In this technology, depending on the physical and chemical properties of the compounds to be removed, the temperature, pressure and time can be adjusted. The effectiveness of this technology depends on food composition, moisture content and the nature of the compound to be removed [12, 132]. Viscosity minimizes the diffusion of molecules through the matrix and thus compromises the effect of the process in dry foods [49]. Because honey is liquid or semi-liquid, the process can effectively remove HMF from honey. The technology can be applied to any finished product without any modification of any of the process parameters [11].

## Conclusion and future directions

Multiple factors affect HMF production in honey and other food products. Therefore, determining how to prevent or reduce the formation of HMF remains a challenge. In addition, the determination of the compound’s concentration and the low efficiency of removal methods make it difficult to establish a complete database of HMF concentrations in different monofloral and multifloral honeys. As a constituent of processed foods, HMF has both profoundly adverse and beneficial effects on human and bee health. Some effects of HMF on human health and its carcinogenic as well as anti-carcinogenic properties remain inconclusive, with many studies conducted only at preclinical levels.

## Abbreviation

AAPH: 2,2′-azobis (2-amidinopropane) dihydrochloride
ACF: aberrant crypt foci
AMS: acute mountain sickness
APC: adenomatous polyposis coli
BBB: blood–brain barrier
CAT: catalase
CCFD: central composite face-centered design
CYP2E1: cytochrome P450 2E1
DART: direct analysis in real time
DMF: 2,5-dimethylfuran
DPPH: 2,2-diphenyl-1-picrylhydrazyl
DRP: deoxyribose phosphate
EFSA: European Food Safety Authority
ERK: extracellular signal-regulated kinase
FAP: familial adenomatous polyposis syndrome
FcεRI: express immunoglobulin Fc epsilon receptor type I
FDCA: 2,5-furandicarboxylic acid
FFA: furfuryl alcohol
GPx: glutathione peroxidase
IFNγ: interferon gamma
IgE: immunoglobulin E
IHC: International Honey Commission
IL-4: interleukin 4
H_2_O_2_: hydrogen peroxide
HACE: high-altitude cerebral edema
HAPE: high-altitude pulmonary edema
HbS: sickle hemoglobin
HFCS: high fructose corn syrup
HIF: hypoxia inducible factors
HMF: 5-hydroxymethylfurfural
HMFA: 5-hydroxymethylfuroic acid
HMFG: 5-(hydroxymethyl)-2-furoyl glycine
HPLC-DAD: high-performance liquid chromatography coupled with a diode array detector
MAPK: mitogen activated protein kinase
MAD: malondialdehyde
MEKC: micellar electrokinetic capillary chromatography
NADPH: nicotinamide adenine dinucleotide phosphate
NFκB: nuclear factor kappa B
NOS2: nitric oxide synthase 2
OAT1 and OAT2: organic anion transporters type 1 and 2
OVA: ovalbumin
PAPS: 3′-phosphoadenosine-5′-phosphosulfate
PPARγ: peroxisome proliferator-activated receptor γ
ROS: reactive oxygen species
RSM: response surface methodology
SMF: 5-sulfoxymethylfurfural
SOD: superoxide dismutase
TDI: tolerable daily intake
SULT: sulfotransferase
TdT: terminal deoxynucleotidyl transferase
TOF-MS: time-of-flight mass spectrometry
UHT: ultra-high temperature processing
XO: xanthine oxidase
